# Cognitive rehabilitation in schizophrenia research: a bibliometric and visualization analysis

**DOI:** 10.3389/fpsyt.2024.1509539

**Published:** 2024-12-11

**Authors:** Xiaofeng Shu, Yubin Dai, Juanping Tang, Yi Huang, Rong Hu, Yong Lin

**Affiliations:** ^1^ Rehabilitation Medical Center, Kangci Hospital, Jiaxing, Zhejiang, China; ^2^ The Third School of Clinical Medicine (School of Rehabilitation Medicine), Zhejiang Chinese Medical University, Hangzhou, China; ^3^ College of Acupuncture-Massage and Rehabilitation, Hunan University of Chinese Medicine, Changsha, China

**Keywords:** schizophrenia, cognitive, rehabilitation, bibliometrics, global trends

## Abstract

**Objective:**

Cognitive impairment is notably prevalent among schizophrenic individuals and is acknowledged as one of the core features of the disorder. Despite the proliferation of literature on cognitive rehabilitation treatments for schizophrenia in recent years, there remains a dearth of systematic reviews and selections of research in this area. From a bibliometric perspective, this study aims to analyze and discuss the current state, developmental trends, and potential research hotspots of cognitive rehabilitation in schizophrenia over the past two decades.

**Methods:**

The Core database of Web of Science was utilized to retrieve articles on cognitive rehabilitation in Schizophrenia that were published from 2004 to 2024. Bibliometrics was applied to perform both quantitative and qualitative analyses of authors, institutions, countries, journals, references, and keywords, leveraging tools such as CiteSpace, VOSviewer, and the R software package Bibliometrix.

**Results:**

A total of 2,413 articles were encompassed in this study, comprising 1,774 regular articles and 373 review articles. The United States emerged as the country with the highest productivity and citation counts, engaging in academic collaborations with over 40 nations. This was followed by the United Kingdom and Spain. King’s College London stood out as the leading institution in the field. However, the article with the highest average citation rate was authored by Susan R. McGurk from the Dartmouth Centre for Psychiatric Research in the United States. Schizophrenia Research proved to be the most influential journal in this domain, with its articles being cited over 10,000 times.

**Conclusion:**

This study provides a comprehensive review of research achievements in cognitive rehabilitation for schizophrenia spanning from 2004 to 2024, and outlines global research hotspots and trends with future projections. Currently, methods for cognitive rehabilitation in schizophrenia and neural plasticity in the brain represent the cutting-edge of research. The safety, efficacy, and standardization of virtual reality are poised to emerge as potential future hotspots and trends in research. Additionally, the neurobiological foundations of cognitive remediation therapy constitute an unexplored territory ripe for further investigation.

## 1 Introduction

Schizophrenia is a severe and complex mental health disorder of the brain that profoundly affects individuals, manifesting a range of intricate clinical symptoms. These symptoms encompass not only hallucinations, delusions, emotional blunting, and social withdrawal, but also severe cognitive impairments. Such cognitive deficiencies are particularly prevalent among individuals with schizophrenia and are considered one of the core features of the disorder (1, 2), extensively impacting multiple crucial domains such as memory, attention, executive function, and language processing, posing formidable challenges to the functioning and rehabilitation of patients with schizophrenia spectrum disorders (3, 4).

As an evidence-based intervention, cognitive remediation therapy(CRT) aims to enhance patients’ cognitive abilities through the implementation of systematic training programs (5, 6). It is considered that CRT should be consistently included in clinical guidelines for the treatment of schizophrenia and more widely implemented in clinical practice (7). This approach has increasingly attracted the attention of researchers and clinicians, finding broad application in clinical settings and demonstrating significant potential in promoting the recovery of patients’ cognitive functions (8, 9). The number of publications on CRT for schizophrenia has surged in recent years, with multiple reviews and meta-analyses discussing cognitive remediation in schizophrenia (10–12). However, studies mainly focus on the clinical feasibility, safety and effectiveness of CRT (13–15). Although this provides a solid premise for the clinical popularization of CRT, systematic evaluation studies on the overall research status, trend and mechanism of CRT treatment are still lacking. Therefore, a systematic evaluation of the overall status quo, trend and limitations of CRT research on schizophrenia, and analysis of its hot spots and potential research directions can not only enable relevant personnel to grasp its research progress more clearly, but also provide researchers with new research strategies to better explore the pathological mechanism of the disease process of schizophrenia. It may also be possible to develop drug treatments that provide precise targets.

Bibliometrics, as an interdisciplinary research method, employs mathematical and statistical principles to conduct quantitative analyses of various knowledge carriers, revealing key information such as the distribution characteristics of literature and trends in disciplinary development. It provides important methodological support for scientific research and has now become an indispensable part of bibliographic studies. In the last two decades, with the rapid development of cognitive neuroscience, CRT has emerged as a promising option for the treatment of schizophrenia. In order to more systematically summarize and elaborate the research of CRT in schizophrenia, this study intends to use bibliometrics to conduct an in-depth quantitative analysis of relevant literature published from 2004 to 2024. The aim is to identify key researchers, core publications, and high-frequency vocabulary in this field, thereby comprehensively and systematically presenting the current application status, developmental trends, and potential research hotspots of CRT in schizophrenia. Furthermore, this study also expects to promote continuous progress in the field of CRT by deeply analyzing the existing deficiencies and challenges in current research, ultimately striving to provide more efficient and comprehensive treatment options for schizophrenic patients.

## 2 Materials and methods

### 2.1 Data sources and search strategy

**Figure 1:**
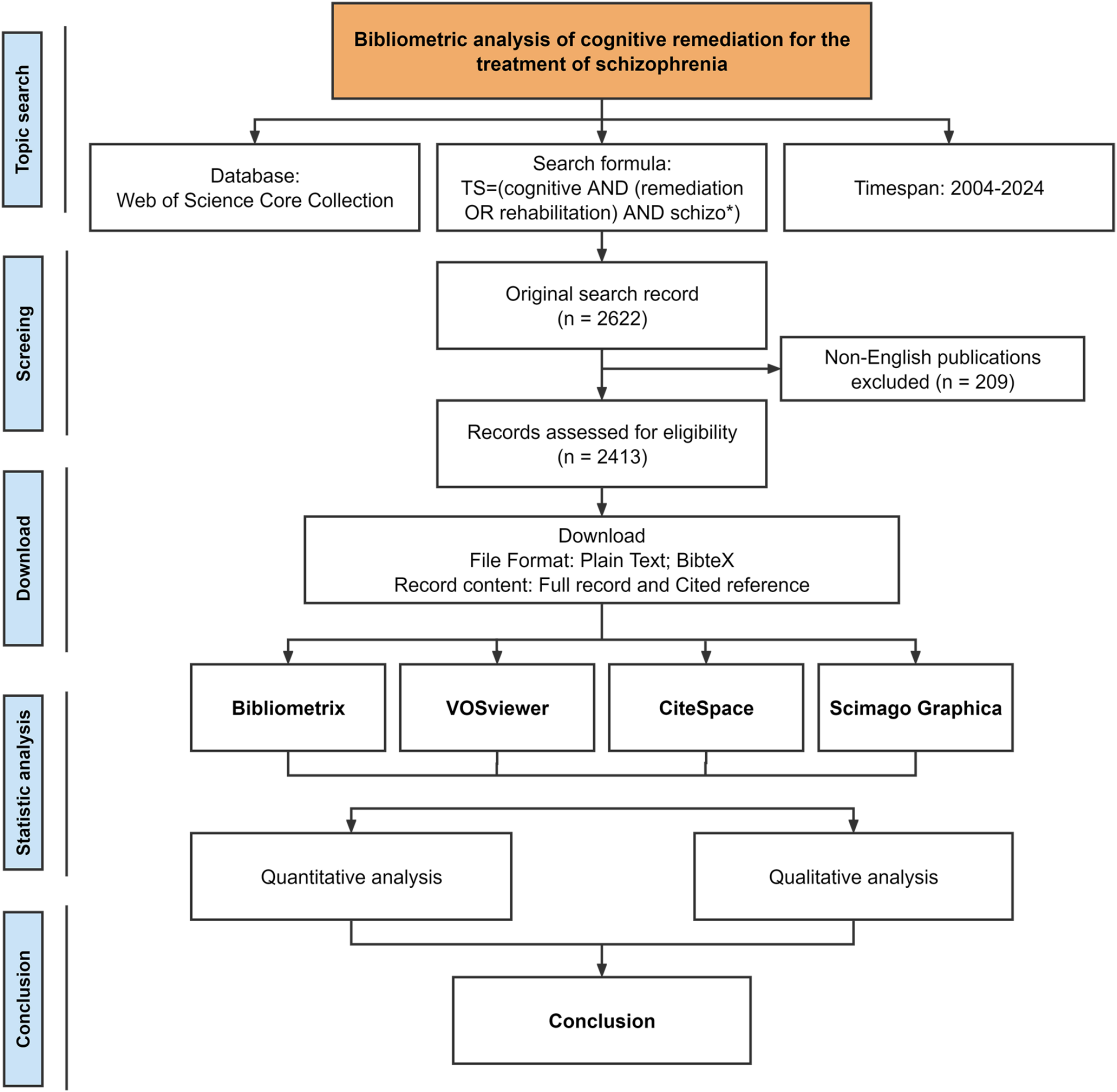
Flow chart of scientometric analysis. *: Used as a wildcard in this search formula, it can represent zero or more characters and is used to expand the search scope.

Web of Science, an independent global citation database developed and maintained by Clarivate Analytics, provides comprehensive coverage of over 12,000 international academic journals and is frequently utilized by researchers (16). To enhance the representativeness and accessibility of the data, we focused on the Web of Science Core Collection. The scientometrics research process is shown in **Figure 1**. We employed the search query “Topic Search = (cognitive AND (remediation OR rehabilitation) AND schizo*)” to retrieve literature from indexes such as SCIE, SSCI, A&HCI, and ESCI, with a time span ranging from January 1, 2004, to August 28, 2024. To facilitate further analysis of the literature content, we restricted the publication language to English. Subsequently, we extracted complete records and cited references from the relevant publications and saved them in Plain Text and BibTeX formats for further investigation.

### 2.2 Data analysis methods

In this study, VOSviewer (version 1.6.19), R software (version 4.1.2), Citespace (version 6.2.R4), and Scimago Graphica (version 1.0.44) were utilized as the primary tools for a multidimensional statistical and visual quantitative analysis of cognitive remediation therapy for schizophrenia. Building on this foundation, we applied the key path method to analyze data collection elements, constructed knowledge maps, utilized co-occurrence graphs to investigate research hotspots over the years, and qualitatively analyzed the developmental relationships among research hotspots. In particular, the R software toolkit “Bibliometrix” was used to categorize the number of relevant publications and citations according to the source, institution, country, author, and other elements through the Biblioshiny application (17). VOSviewer is free software based on the Java programming language that includes text mining functionality (18). In this paper, it was used to construct national citation networks, institutional co-authorship networks, and keyword co-occurrences and combined with Scimago Graphica for graph beautification (19). Citespace is an information visualization software based on the theory of citation analysis, focusing on prospective data in the scientific literature (20). We used Citespace to detect keywords with high citation bursts and plot a keyword clustering timeline.

## 3 Results

### 3.1 Overview of publications

**Figure 2:**
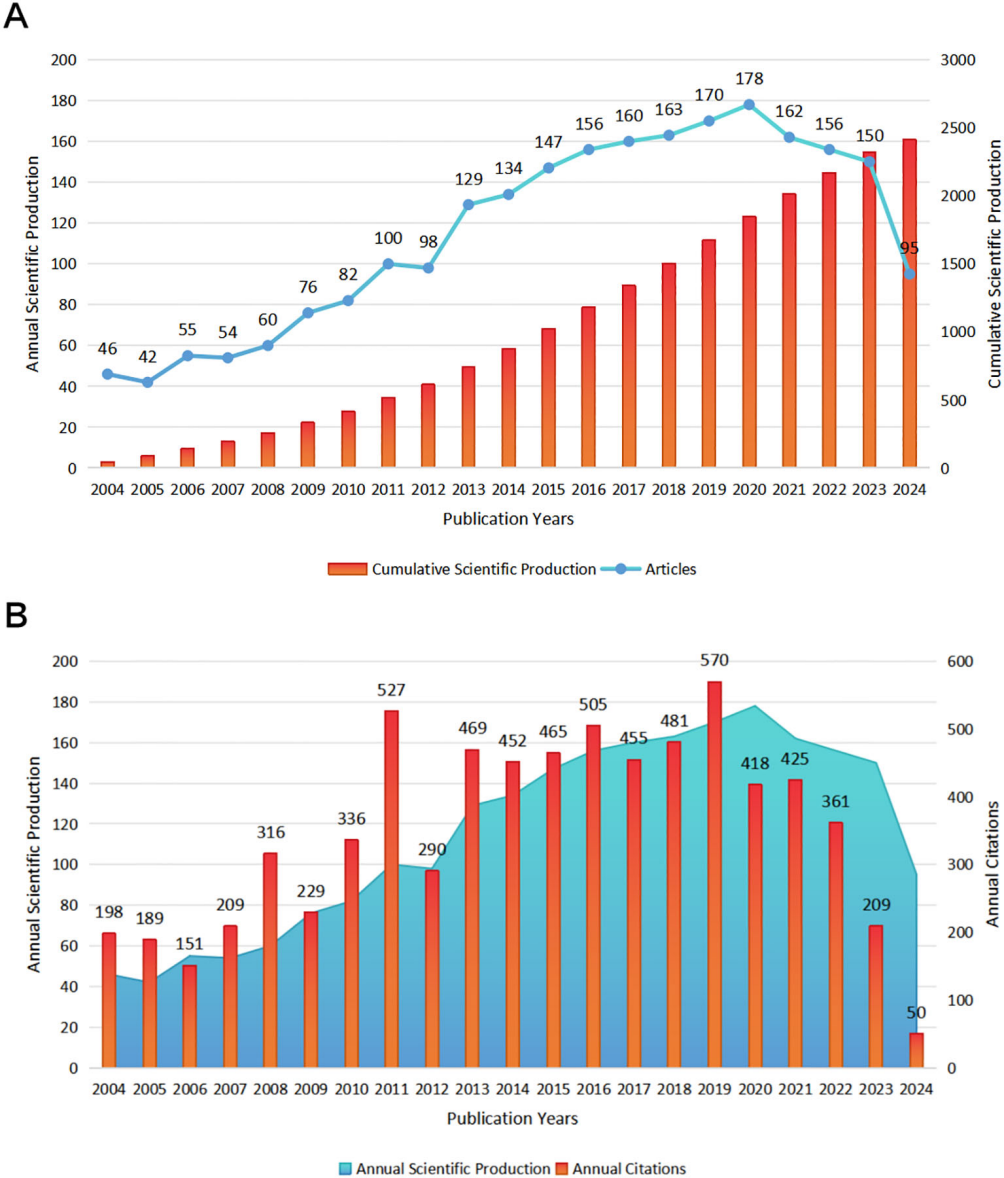
**(A)** Annual and cumulative research results; **(B)** Annual research results and citations.

A total of 2413 publications related to cognitive remediation therapy for schizophrenia were included, with 1774 being regular articles and 373 being review articles. Over the past 20 years, there has been a steady increase in the number of relevant papers, with an annual growth rate of 3.69% (**Figure 2A**). Specifically, from 2004 to 2013, the average annual publication count was 74.2, showing a slow growth trend. However, as mental illnesses have gained increasing attention and the potential of cognitive remediation therapy in cognitive rehabilitation for schizophrenia has been recognized, this field has received widespread attention. In the last decade, the number of papers has shown an exponential growth trend, with the total publications increasing from 876 in 2014 to 2413 in 2024, averaging over 150 publications per year. We further analyzed the annual citation frequency. The average citations per document for the publications were 31.23 (**Figure 2B**). Notably, the mean total citation per article in 2004 was as high as 90.52, indicating significant attention from researchers towards publications in that year (**Figure 2B**). As of 2020, the overall citation volume has generally shown an inverse trend with publications.

### 3.2 Analysis of countries or regions

**Figure 3:**
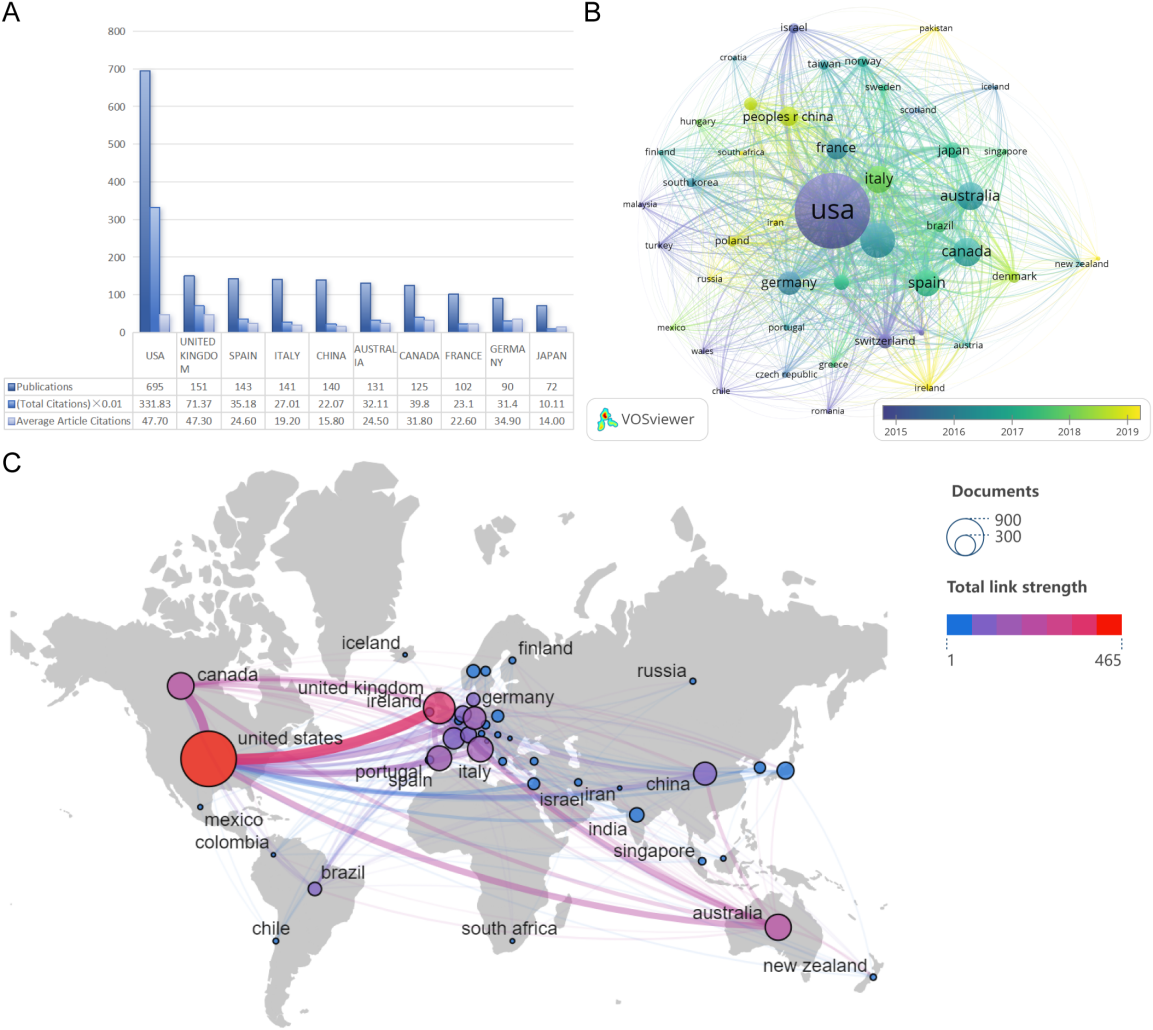
**(A)** Country distribution of corresponding authors; **(B)** bibliographic coupling countries overlay visualization **(C)** National cooperation.

A total of 54 countries/regions have published articles in this research field. However, when the minimum document count was set to 100, only 8 countries/regions met the threshold. As shown in **Figure 3A**, the country with the highest productivity and citation count is the United States (n=695; total citations=33,183; average article citations=47.7), accounting for 28.8% of the total included papers, followed by the United Kingdom (n=151; total citations=7,137; average article citations=47.3) and Spain (n=143; total citations=3,980; average article citations=31.8). **Figure 3B** presents a visual overlay of bibliographic coupling countries, where the color of the circles indicates the average publication year for each country/region in this field within the time-overlapping network. It is evident that the United States is an early pioneer in research on cognitive remediation therapy for schizophrenia, while countries such as China, Russia, and Poland have relatively newer research in this field. Furthermore, the United States is the most active country in this field, with collaborative relationships established with 40 countries (**Figure 3C**). Among them, there have been 66 co-authorships with the United Kingdom over the past 20 years.

### 3.3 Institutional analysis

**Figure 4:**
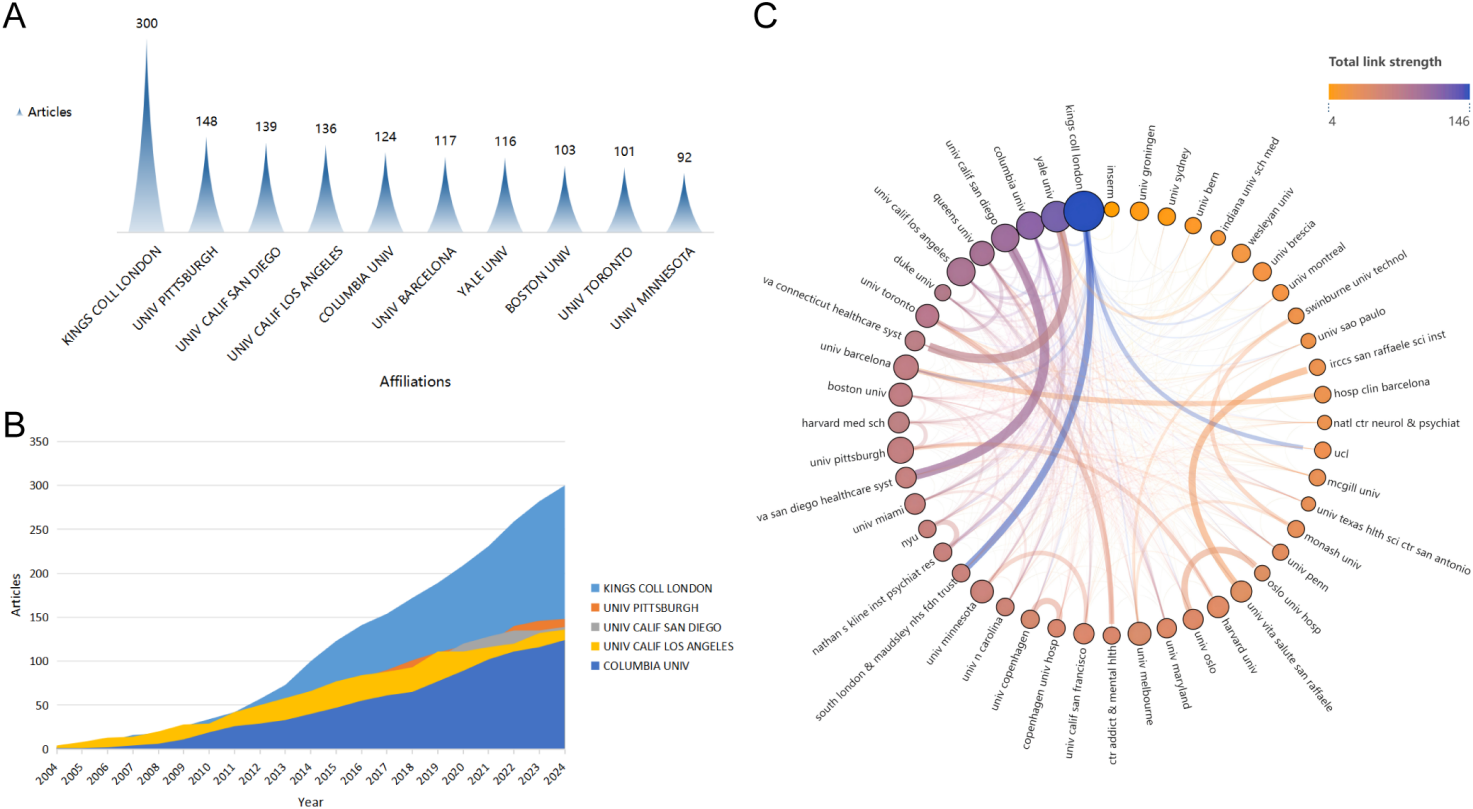
**(A)** The top 10 institutions in the number of publications; Top 5 institution’s production over time; Top 5 institution’s production over time; **(C)** Network of institutional co-authors.

Research on cognitive remediation therapy for schizophrenia has been conducted by approximately 2947 organizations worldwide. As shown in **Figure 4A**, among the top 10 institutions in terms of productivity, 7 are from the United States, while the remaining 3 are located in the United Kingdom, Spain, and Canada. King’s College London has long been a leader in this field, with a total of 300 related publications, ranking first (**Figure 4B**). It is followed by the University of Pittsburgh and the University of California, San Diego. To further investigate collaboration among institutions, we conducted a co-authorship analysis on all publications. With a threshold of at least 20 publications, 48 institutions were included in the institutional co-authorship network (**Figure 4C**). Among them, the top three in total link strength (TLS) are King’s College London (TLS=146), Yale University (TLS=123), and Columbia University (TLS=112).

### 3.4 Author analysis

**Figure 5:**
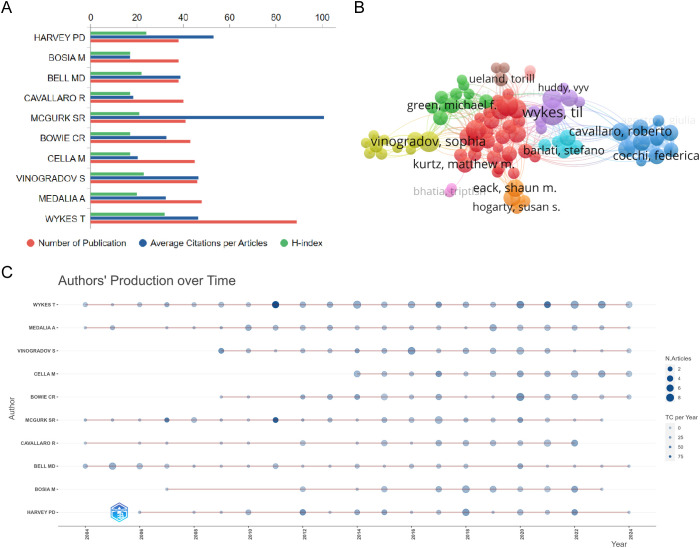
**(A)** The number of publications of the top 5 authors, the average number of citations per article and the H-index; **(B)** Co-author analysis **(C)** Authors’ production over time. The circle size represents the number of references (N. references), and the shade of the color represents the total number of citations (TC).

A total of 9679 authors have participated in research related to cognitive remediation therapy for schizophrenia. Til Wykes from the Institute of Psychiatry, Psychology & Neuroscience at King’s College London is the most productive author, with 89 publications and an H-index of 32 (**Figure 5A**). However, the author with the highest average citation per article is Susan R. McGurk from the Dartmouth Psychiatric Research Center in the United States. She has published 41 related studies, with an H-index of 21 and an average citation per article exceeding 100. With a threshold of at least 10 publications and after removing isolated, unconnected nodes, 84 authors were included in the co-author analysis, divided into 10 closely connected clusters (**Figure 5B**). Among them, Roberto Cavallaro from Vita-Salute San Raffaele University has the closest international collaboration (TLS=217). Additionally, the production timelines of the top 10 authors by publication count indicate that more and more outstanding researchers have been participating in this field in the last decade.

### 3.5 Journal analysis

We identified 433 journals using the R package “bibliometrix”, among which 41 journals met the threshold of at least 10 publications. **Table 1** summarizes the top 10 most relevant journals and lists their related information. Among them, 6 journals are in the first quartile (Q1) of the Journal Citation Reports (JCR). Five publishers are from the United Kingdom, three from the Netherlands, and the other two from the United States and Switzerland, respectively. After sorting by the number of publications, “Schizophrenia Research” tops the list with 281 publications, while “Schizophrenia Bulletin” (n=157) and “Psychiatry Research” (n=118) rank second and third, respectively. In terms of citations, “Schizophrenia Research” (10,243 citations) also ranks first, followed by “Schizophrenia Bulletin” (7,267 citations) and “American Journal of Psychiatry” (7,190 citations).

**Table 1:** Top 10 most relevant journals.

Rank		Journal	Article	Total Citations	JCR (2023)	IF (2023)	Publisher
1		Schizophrenia Research	281	10243	Q1	3.6	ELSEVIER
2		Schizophrenia Bulletin	157	7267	Q1	5.3	OXFORD UNIV PRESS
3		Psychiatry Research	118	3123	Q1	4.2	ELSEVIER IRELAND LTD
4		Frontiers in Psychiatry	89	1225	Q2	3.2	FRONTIERS MEDIA SA
5		Psychological Medicine	59	2071	Q1	5.9	CAMBRIDGE UNIV PRESS
6		BMC Psychiatry	43	729	Q2	3.4	BMC
7		Psychiatric Rehabilitation Journal	42	995	Q3	1.8	EDUCATIONAL PUBLISHING FOUNDATION-AMERICAN PSYCHOLOGICAL ASSOC
8		European Psychiatry	40	742	Q1	7.2	CAMBRIDGE UNIV PRESS
9		Journal of Psychiatric Research	39	1282	Q1	3.7	PERGAMON-ELSEVIER SCIENCE LTD
10		Schizophrenia Research-Cognition	36	400	Q2	2.3	ELSEVIER

### 3.6 Highly cited literature analysis

**Table 2** lists the top 10 most cited publications, all of which have been cited more than 500 times. Among these articles, 5 are reviews, 3 are meta-analyses, and 2 are clinical studies. The most cited article titled “The MATRICS Consensus Cognitive Battery, Part I: Test Selection, Reliability, and Validity” published in the American Journal of Psychiatry in 2008 (21). This study aims to conduct a broad scientific assessment of interventions and develop standard tools for evaluating cognitive changes in clinical trials of cognitive enhancement therapies for schizophrenia. A 2016 review published in The Lancet titled “Schizophrenia” ranked fifth in the number of citations, despite its relatively short publication time. It provides a detailed elaboration on eight aspects of schizophrenia, including clinical manifestations, epidemiology, pathology, and treatment (22). The three meta-analyses all show that cognitive remediation is beneficial for patients with schizophrenia and can also improve functional outcomes when combined with psychiatric rehabilitation, providing strong evidence for the clinical application and promotion of cognitive remediation (23–25).

**Table 2:** Top 10 articles in total citations.

Rank	Year	First author	Title	Journal	Total citations
1	2008	Keith H Nuechterlein	The MATRICS Consensus Cognitive Battery, part 1: test selection, reliability, and validity	American Journal of Psychiatry	1716
2	2011	Anne-Kathrin J Fett	The relationship between neurocognition and social cognition with functional outcomes in schizophrenia: a meta-analysis	Neuroscience And Biobehavioral Reviews	1327
3	2011	Til Wykes	A meta-analysis of cognitive remediation for schizophrenia: methodology and effect sizes	American Journal of Psychiatry	1170
4	2004	Michael F Green	Longitudinal studies of cognition and functional outcome in schizophrenia: implications for MATRICS	Schizophrenia Research	1089
5	2016	Michael J Owen	Schizophrenia	Lancet	894
6	2004	Anabel Martínez-Arán	Cognitive function across manic or hypomanic, depressed, and euthymic states in bipolar disorder	American Journal of Psychiatry	872
7	2007	Susan R McGurk	A meta-analysis of cognitive remediation in schizophrenia	American Journal of Psychiatry	834
8	2005	Peter Milev	Predictive values of neurocognition and negative symptoms on functional outcome in schizophrenia: a longitudinal first-episode study with 7-year follow-up	American Journal of Psychiatry	706
9	2015	René S Kahn	Schizophrenia	Nature Reviews Disease Primers	701
10	2007	Vikram Patel	Treatment and prevention of mental disorders in low-income and middle-income countries	Lancet	571

### 3.7 Keyword analysis

**Figure 6:**
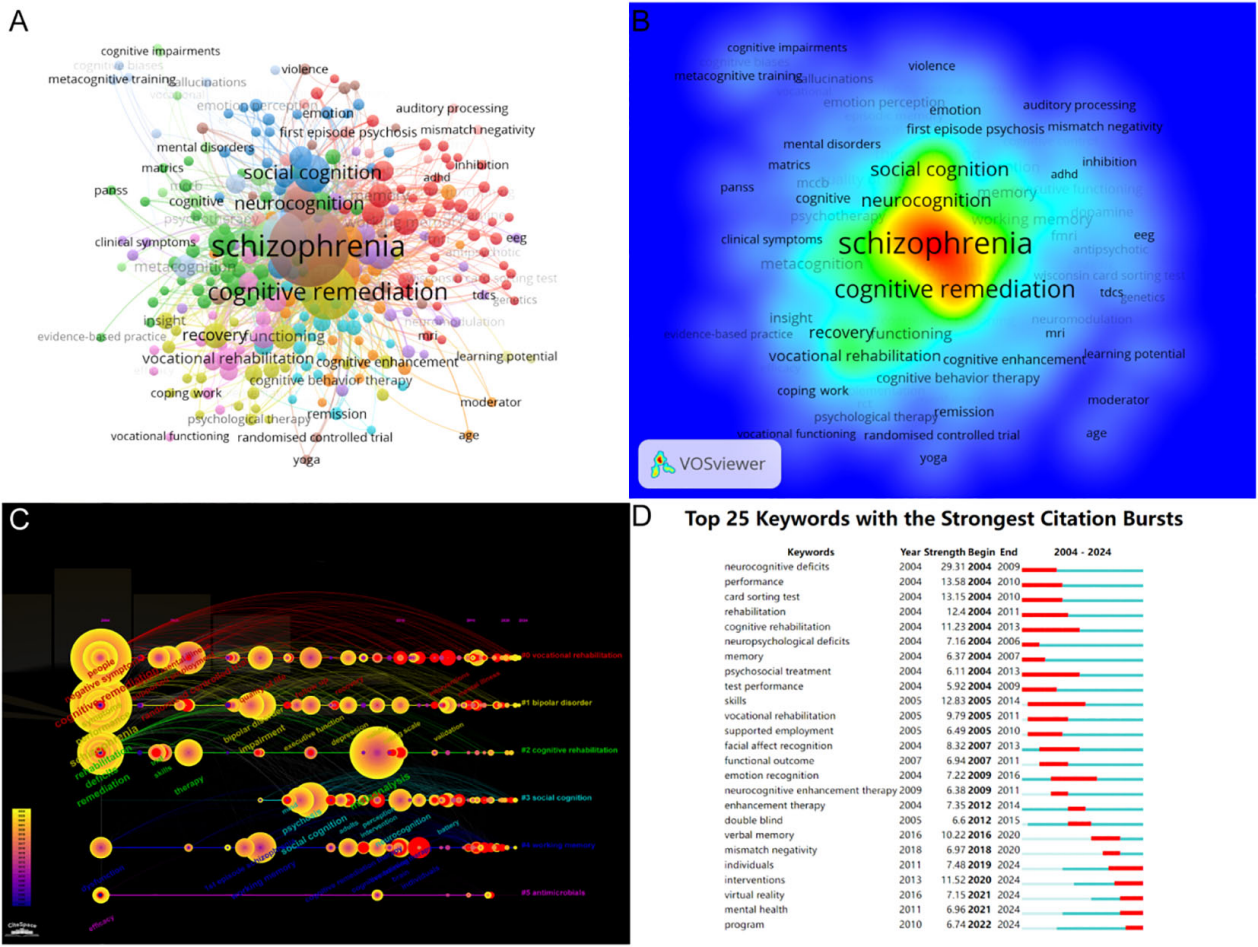
**(A)** keyword co-occurrence network visualization **(B)** keyword density map **(C)** keyword clustering timeline. **(D)** Top 25 keywords with the strongest citation bursts.

Keyword co-occurrence analysis identifies emerging research themes and their relevance by counting the frequency and co-occurrence of keywords in the literature. With a threshold of at least five occurrences, 274 out of 2995 keywords met the criteria. Each node represents one keyword, the size of the node is proportional to the frequency, the distance of the node is proportional to the relevance, and keywords with similar characteristics form a cluster. The keywords were clustered into 13 clusters based on TLS and orientation, with the same color representing the same cluster (**Figure 6A**). The largest cluster (red) contained 45 nodes, represented by the keywords “bdnf,” “dopamine,” “prefrontal cortex,” “plasticity,” “white matter,” and “neuroimaging.” The density map showed that the most frequent keywords were “schizophrenia” (n=1,102), “cognitive remediation” (n=447), and “cognition” (n=359) which were consistent with the study field (**Figure 6B**). The keyword cluster timeline can showcase the time periods and development trends of different research keywords. According to the special algorithm of CiteSpace, the keywords related to cognitive remediation therapy for schizophrenia are divided into five major clusters: “vocational rehabilitation,” “bipolar disorder,” “cognitive rehabilitation,” “social cognition,” and “working memory” (**Figure 6C**). Additionally, CiteSpace can identify major themes by detecting burst keywords within specific time periods. The keyword “cognitive rehabilitation” (2005–2014) received the most sustained attention, while “cognitive deficits” (strength=29.31) had the highest burst strength (**Figure 6D**). It is worth noting that the emergence of keywords such as “individuals,” “interventions,” “virtual reality,” “mental health,” and “program” has persisted until now, indicating future research directions.

## 4 Discussion

This study focuses on the research status and progress of schizophrenia in the field of cognitive therapy. Here, we use bibliometrics methods to conduct an in-depth analysis of the growth trend of cognitive repair research in schizophrenia from 2004 to 2024, and comprehensively understand the global research trends and research hotspots in this field.

### 4.1 Global trends in cognitive rehabilitation research in schizophrenia

Our analysis indicates that from 2004 to 2024, there has been a steady increase in the annual publication output within the field of cognitive rehabilitation related to schizophrenia, with an annual growth rate of 3.69%. Since 2014, the number of related papers published has significantly surged, exceeding 150 per year. In stark contrast to this thriving publication trend, the citation counts of these papers have shown a declining pattern. Notably, the United States, the United Kingdom, and Spain are major contributors to research in this field, having published 989 papers, accounting for 40.99% of the total literature. Furthermore, the United States has established research partnerships with 40 countries globally, playing a pivotal role in international collaboration. Professor Susan R. McGurk from the Dartmouth Psychiatric Research Center in the United States stands at the forefront of this domain. She has published 41 relevant studies, boasts an H-index of 21, and her papers have been cited over 100 times each on average. Given its leading position in the field and the high quality of its content, Schizophrenia Research is the preferred journal for researchers to submit their work.

The number of citations, as a key indicator for measuring the academic impact of publications, uncovered the research themes and focal points in this field. Studies by Anabel Martínez-Arán (26) and Peter Milev (27) on cognitive function in different states of bipolar disorder and other psychiatric diseases and their relationship with negative symptoms further confirmed the importance of cognitive impairments. These studies were among the top ten most cited literature. The European Psychiatric Association listed cognitive remediation as the highest recommended intervention in its guidelines for the treatment of schizophrenia (28). Keith H. Nuechterlein’s article published in the American Journal of Psychiatry in 2008, with 1,716 citations, became the gold standard for measuring cognitive changes in schizophrenia, providing important consensus for related clinical trials of cognitive remediation treatments (21). Subsequently, basic and translational research on cognitive impairments in schizophrenia received widespread support, driving advancements in cognitive measurement, drug development, and behavioral intervention methods. This promoted patient-centered, clinically effective, and continuously optimized cognitive healthcare services (29), ensuring the effectiveness and reliability of treatments.

### 4.2 Research hotspots and development predictions

High-frequency keywords are commonly used to accurately reveal the primary research directions and hotspots within a specific field during a certain period. Combined with burst detection, they can assist researchers in swiftly capturing research trends from numerous studies. From keyword co-occurrence analysis, we have identified “dopamine,” “prefrontal cortex,” “plasticity,” “white matter,” and “neuroimaging” as recent hot topics, which are likely to remain at the forefront of research in the short term. The timeline of keyword clusters illustrates the time periods and development trends of different research keywords, with the emergence of keywords such as “individuals,” “interventions,” “virtual reality,” “mental health,” and “program” persisting to the present, indicating future research directions.

#### 4.2.1 Neuroplasticity plays a pivotal role in cognitive remediation for schizophrenia: a potential research trend and hot direction

Jaskirat Singh posits that schizophrenia is a profound and intricate mental disorder characterized by spatio-temporal cortical abnormalities, intricately linked to alterations in brain system functionality (8). Abnormal cortical thickness at different stages of schizophrenia (30), such as reduced thickness of the right inferior frontal gyrus (IFG) and bilateral insular cortex extending into the superior temporal gyrus (STG) (31). Cognitive remediation therapy has been shown to enhance cognitive function and induce neuroplasticity in individuals with schizophrenia, as demonstrated by changes in functional connectivity within regions such as the prefrontal cortex, temporal areas, basolateral amygdala, parietal lobe, and occipital lobe during tasks involving working memory or executive function following CRT (32–34). However, different CRT methodologies may yield varied outcomes.

Currently, cognitive remediation approaches for schizophrenia mainly encompass pharmacological treatment (35), physical therapy (36), cognitive rehabilitation training and cognitive behavioral therapy (CBT) (37), as well as nutrition and lifestyle optimization (38). Antipsychotic medications, as the cornerstone of treatment, not only effectively alleviate psychotic symptoms such as hallucinations and delusions but also facilitate the recovery of cognitive functions to some extent (39, 40). These medications primarily act by blocking dopamine D2 receptors and may improve cognitive function through mechanisms involving neurotransmitter systems and enhancing neuroplasticity (41, 42).

Mainstream physical therapies include repetitive transcranial magnetic stimulation (rTMS) and transcranial electrical stimulation (tDCS/tACS/tRNS) (43). Studies have demonstrated that rTMS and tDCS, as novel non-invasive neuromodulation techniques, are effective in improving cognitive function in schizophrenic patients (44, 45). Different types of transcranial electrical stimulation act on specific brain regions through various mechanisms, modulating neural activity and promoting the recovery of cognitive function by altering membrane potentials, neurotransmitter levels, and neural oscillations (46, 47).

Computerized cognitive remediation therapy (CCRT), as a novel rehabilitation technique that integrates neuropsychological and cognitive rehabilitation theories, has been widely applied (48, 49). CBT improves emotions and behaviors by changing negative thought patterns, helping patients identify and alter negative thoughts, thereby enhancing cognitive function and emotional regulation (50). These therapeutic methods are closely related to promoting neuroplasticity (51, 52), and their mechanisms may involve functional modulation of key brain regions such as the prefrontal cortex, amygdala, and cerebellum (53, 54).

Research indicates that CRT responsiveness is associated with baseline measurements of frontal and temporal cortical thickness, and improvements in language memory may be closely linked to cortical thickness (CTh) in certain regions of the frontal and temporal lobes (55). CRT has been shown to significantly increase the volume of the right hippocampus and induce cognitive improvements through hippocampal plasticity (56). Additionally, CRT can enhance myelin function in the left posterior cerebellar lobe of patients with schizophrenia, thereby improving cognitive function (57). Therefore, summarizing the effects of different cognitive remediation programs on brain activity holds great potential benefits for developing personalized rehabilitation plans in the future (58).

#### 4.2.2 Virtual reality: an emerging approach for cognitive remediation in schizophrenia

Additionally, keyword prominence is helpful for researchers to grasp the research hotspot quickly. In this study, keywords such as “individual,” “intervention,” “virtual reality,” “mental health,” and “project” consistently appear. This indicates that cognitive remediation is one of the mainstream therapies for schizophrenia. With advancements in science and technology, virtual reality (VR) has increasingly garnered attention as an immersive therapeutic tool in the field of mental health (59, 60). A recent meta-analysis report, including 11 different studies, pointed out that VR-based CR interventions had a significant positive impact on cognitive function (61–63). Therefore, the safety, efficacy, and standardization of VR may be future research hotspots and trends. Virtual reality provides multisensory embodied experiences that may engage brain network function more effectively than standard cognitive training systems.

### 4.3 Limitation

The biological foundations of cognitive impairments in schizophrenia can provide precise and novel evidence for clinical efficacy and the development of therapeutic drugs. Recent studies have highlighted the distinct roles of neuroinflammation and kynurenine pathway metabolites in inducing cognitive deficits, which are contingent upon cognitive reserve and predictive of post-rehabilitation outcomes (64). There is a link between the anti-inflammatory effects induced by rTMS and improvements in psychopathology, yet the generalizability of these results is constrained by analysis heterogeneity and insufficient power (65). Unfortunately, current research primarily focuses on clinical safety and efficacy, and while it touches upon the significant impact of neural plasticity changes in the brain, it largely remains at the structural level, with limited exploration into the underlying neurobiological changes and mechanisms—a domain ripe for further investigation.

In addition, this study has certain limitations. Firstly, it only included English articles indexed in the WoSCC database, but the majority of high-quality studies were covered, which does not affect the overall trend of the results. Secondly, recently published high-quality studies may not have been fully evaluated due to delayed citations. Nevertheless, this study still aids researchers in gaining a deeper understanding of the development, trends, and frontiers of cognitive remediation treatment for schizophrenia and identifying areas that require further research in the future.

## 5 Conclusion

This study provides a comprehensive review of research achievements in cognitive rehabilitation for schizophrenia spanning from 2004 to 2024, and outlines global research hotspots and trends with future projections. Currently, methods for cognitive rehabilitation in schizophrenia and neural plasticity in the brain represent the cutting-edge of research. The safety, efficacy, and standardization of virtual reality are poised to emerge as potential future hotspots and trends in research. Additionally, the neurobiological foundations of cognitive remediation therapy constitute an unexplored territory ripe for further investigation.

## Data Availability

The original contributions presented in the study are included in the article/supplementary material. Further inquiries can be directed to the corresponding authors.
